# Transcranial static magnetic field stimulation of the primary motor cortex in essential tremor: a randomized pilot study

**DOI:** 10.1038/s41531-025-01182-x

**Published:** 2025-11-26

**Authors:** Daniele Urso, Mariana H. G. Monje, Beatriz Fernández-Rodríguez, Lydia Vela-Desojo, Antonio Oliviero, Guglielmo Foffani, Claudia Ammann

**Affiliations:** 1 HM CINAC (Centro Integral de Neurociencias Abarca Campal), Hospital Universitario HM Puerta del Sur, HM Hospitales, Madrid, Spain; 2 Centre for Neurodegenerative Diseases and the Aging Brain, Department of Clinical Research in Neurology, University of Bari ‘Aldo Moro, Pia Fondazione Cardinale G. Panico, 73039 Tricase, Italy; 3 Instituto de Investigación Sanitaria HM Hospitales, Madrid, Spain; 4 Hospital Regional Universitario de Málaga, Málaga, Spain; 5 Hospital Universitario Fundación Alcorcón, Alcorcón, Madrid, Spain; 6 FENNSI Group, Hospital Nacional de Parapléjicos, Toledo, Spain; Instituto de Investigación Sanitaria de Castilla-La Mancha (IDISCAM), Toledo, Spain; 7 CIBERNED, Instituto de Salud Carlos III, Madrid, Spain; 8 Reina Sofia Foundation Alzheimer Center, CIEN Foundation, ISCIII, Madrid, Spain; 9 Facultad HM de Ciencias de la Salud de la Universidad Camilo José Cela, Madrid, Spain; 10 Present Address: Ken and Ruth Davee Department of Neurology, Northwestern University, Feinberg School of Medicine, Chicago, IL USA

**Keywords:** Neurophysiology, Neurological disorders

## Abstract

Alterations in the motor cortex may contribute to essential tremor (ET) pathophysiology. Transcranial static magnetic field stimulation (tSMS), a non-invasive and portable technique, reduces corticospinal excitability, but its therapeutic potential for ET remains untested. This randomized, double-blind pilot study (clinicaltrials.gov#: NCT03780426, registered on December 19, 2018) investigated the acute effects of tSMS applied to the primary motor cortex in 27 ET patients (mean age: 66.9 ± 12.1 years). Patients received a 30-min tSMS session on either the left (*n* = 13) or right (*n* = 14) hemisphere. Postural tremor was assessed by accelerometry, and kinetic tremor by part B of the Clinical Rating Scale for Tremor. Twenty-three patients were included in the final analysis for primary and secondary outcomes. Bayesian analysis showed moderate evidence for a bilateral reduction in postural and rest tremor following unilateral tSMS, with anecdotal reduction of kinetic tremor and moderate evidence of absence of change in tremor frequency. These preliminary findings warrant further studies to assess tSMS as a therapeutic option for ET.

## Introduction

Essential tremor (ET), a complex neurological syndrome, is one of the most prevalent movement disorders, primarily characterized by involuntary rhythmic movements that can become debilitating for daily tasks^1,2^. Despite its frequent occurrence, the understanding of its underlying pathophysiology remains incomplete. In addition, up to 50% of ET patients find current medication unsatisfactory^3,4^. This limited success underscores the need for alternative therapies. Stereotactic interventions targeting the thalamus such as deep brain stimulation^5^, thalamotomy with radiosurgery^6^, gamma knife^7^, or high-intensity focused ultrasound (FUS)^8^ have shown clinical efficacy reducing tremor. Yet, these procedures are not suitable for all patients due to contraindications or aversion to invasive treatments.

Non-invasive brain stimulation (NIBS) techniques may provide a safe and potentially effective alternative for managing ET^9–12^. Nevertheless, most NIBS therapies require daily hospital visits. Portable and user-friendly NIBS methods, such as transcranial static magnetic field stimulation (tSMS), offer an appealing solution for safe long-term, home-based treatments^13–15^. tSMS involves applying a relatively strong neodymium magnet to the scalp, producing a static magnetic field that, unlike transcranial magnetic stimulation (TMS), does not induce an electric field in the underlying brain tissue^16,17^. This approach reduces corticospinal excitability and modulates corticostriatal activity when applied over cortical motor areas after a single application of 10–30 min^17–27^. Thus, tSMS could be a low-cost therapeutic option, as already explored in conditions like levodopa-induced dyskinesias^13^, stroke^28^ and amyotrophic lateral sclerosis (ALS)^14,15^. The hyperkinetic nature of ET also makes it a compelling candidate for tSMS. ET is linked to altered oscillatory activity in the cerebello-thalamo-cortical network^29–38^ and despite differing opinions, several studies have attributed a pathophysiological role to the motor cortex in ET^37–40^. However, although a few studies have utilized NIBS techniques over the cortex to alleviate tremor^9–12^, the effects of cortical tSMS on tremor remain unexplored.

To address this gap, we conducted a randomized, double-blind pilot study to test the hypothesis that tSMS of the primary motor cortex reduces tremor in ET patients. We studied 27 patients (66.9 ± 12.1 years) of which 14 received tSMS of the left hemisphere and 13 of the right hemisphere. The primary outcome was the change from baseline in postural tremor amplitude in the hand opposite the tSMS-treated hemisphere, as measured by accelerometry immediately after tSMS. Secondary outcome measures included: (i) change from baseline in postural tremor amplitude 15 min after treatment; (ii) change from baseline in rest tremor amplitude; (iii) change from baseline in postural tremor frequency; (iv) change from baseline in kinetic tremor, as assessed by the drawing part of Clinical Rating Scale for Tremor (CRST) part B. Methods and results are reported according to CONSORT guidelines^41^.

## Results

### Patients and baseline characteristics

**Fig. 1:**
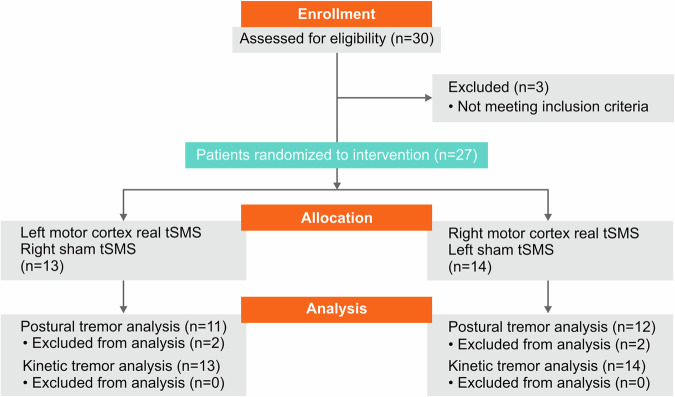
CONSORT flow diagram. The flow diagram shows the participant flow through each stage of the randomized trial (left and right motor cortex tSMS patient group). The different phases are enrollment, allocation, and data analysis. The analysis refers to the primary and secondary outcomes. Tremor recordings were not conducted for all patients, resulting in missing values for four patients.

Twenty-seven patients (13 females; mean age ± standard deviation: 66.9 ± 12.1 years) with definite ET participated in the study (Fig. 1). Average disease duration was 25.9 ± 17.0 years and average total CRST score was 41.3 ± 15.7. All patients exhibited bilateral action tremor of the upper limbs. Six out of 27 patients (22.2%) exhibited rest tremor, and 14 (51.9%) exhibited mild-to-moderate axial tremor. Detailed demographic and clinical data are provided in Table 1.

**Table 1:** Demographic and clinical data at baseline

	All patients	Left tSMS group	Right tSMS group	Left vs. right tSMS group comparisons
Sample size, n	27	13	14	
Age, years ± SD	66.9 ± 12.1	64.6 ± 13.5	68.9 ± 10.7	*BF*_*10*_ = 0.49; *p* = 0.37
Gender, %females	48.2	53.8	42.9	*BF*_*10*_ = 0.40; *p* = 0.59
Disease duration, years ± SD	25.9 ± 17.0	23.5 ± 15.4	28.4 ± 19.0	*BF*_*10*_ = 0.44; *p* = 0.49
CRST total score	41.3 ± 15.7	40.3 ± 16.0	42.2 ± 16.1	*BF*_*10*_ = 0.37; *p* = 0.76
On anti-tremor treatment, %number of patients	23 (85.2%)	12 (92.3%)	11 (78.6%)	*-*

Values reported as mean ± standard deviation (SD). BF and *p* values were assessed with independent samples *t* tests.

*BF* Bayes Factor, *tSMS* transcranial static magnetic field stimulation, *CRST* Clinical Rating Scale for Tremor.

### Clinical efficacy: primary and secondary outcomes

**Fig. 2:**
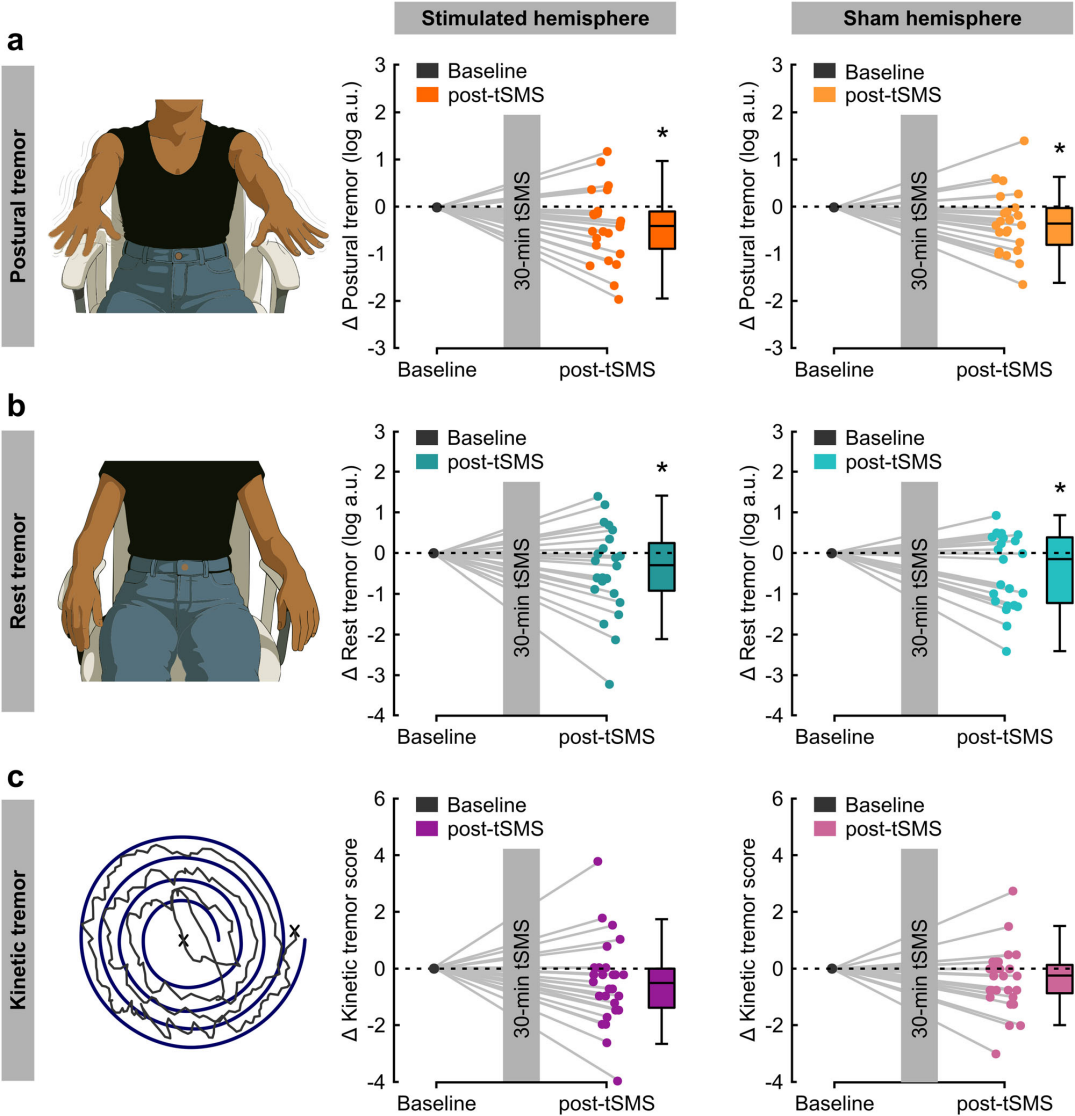
Main results on postural, rest and kinetic tremor. **a** Postural tremor amplitude represented as log-transformed total power values normalized to baseline displayed moderate evidence of reduction immediately after transcranial static magnetic field stimulation (tSMS) compared to baseline (primary outcome; three-way ANOVA, TIME, F(1,21) = 11.3, *p* = 0.003, BF_incl_ = 6.1, η²p = 0.35). The average tremor reduction was similar in the hand contralateral (–60.9%) and in the hand ipsilateral (–51.8%) to the stimulated hemisphere (TIME x SIDE, F(1,21) = 0.1, *p* = 0.72, BF_incl_ = 0.31, η²p = 0.006). **b** Rest tremor amplitude represented as log-transformed total power values normalized to baseline displayed moderate evidence of reduction immediately after tSMS compared to baseline (secondary outcome; three-way ANOVA, TIME, F(1,21) = 4.9, *p* = 0.038, BF_incl_ = 7.1, η²p = 0.19). The average tremor reduction in the hand contralateral (-61.6%) and in the hand ipsilateral (–63.8%) to the stimulated hemisphere (TIME x SIDE, F(1,21) < 0.1, *p* = 0.86, BF_incl_ = 0.28, η²p = 0.001). **c** Kinetic tremor normalized to baseline displayed inconclusive evidence of reduction 5 min after tSMS compared to baseline (secondary outcome; three-way ANOVA, TIME, F(1,25) = 3.6, *p* = 0.068, BF_incl_ = 1.2, η²p = 0.13; TIME x SIDE, F(1,25) = 0.6, *p* = 0.43, BF_incl_ = 0.30, η²p = 0.025). *, BF ≥ 3.

Postural tremor amplitude immediately after tSMS compared to baseline (primary outcome) displayed moderate evidence of reduction (three-way ANOVA, TIME, F(1,21) = 11.3, *p* = 0.003, BF_incl_ = 6.1, η²p = 0.35; Fig. 2a). Interestingly, the average tremor reduction was similar in the hand contralateral (-60.9%) and in the hand ipsilateral (–51.8%) to the stimulated hemisphere (TIME x SIDE, F(1,21) = 0.1, *p* = 0.72, BF_incl_ = 0.31, η²p = 0.006; Fig. 2a). There was inconclusive evidence that stimulation of the right hemisphere might be more effective than stimulation of the left hemisphere (TIME x SIDE x STIMULATION, F(1,21) = 5.0, p = 0.036, BF_incl_ = 1.2, η²p = 0.19). At 15 min after tSMS compared to baseline (secondary outcome) there was no convincing evidence of presence or absence of an effect TIME, F(1,21) = 3.2, *p* = 0.090, BF_incl_ = 0.71, η²p = 0.13; Supplementary Fig. 1a, suggesting that postural tremor was already returning toward baseline values.

Postural tremor frequency (secondary outcome) displayed moderate evidence of absence of change (three-way ANOVA, TIME, F(1,21) = 0.5, *p* = 0.49, BF_incl_ = 0.26, η²p = 0.023).

Rest tremor amplitude (secondary outcome) was, as expected, at least one order of magnitude smaller than postural tremor amplitude, but it was similarly responsive to tSMS. Namely, it displayed (i) moderate evidence of reduction immediately after tSMS compared to baseline (three-way ANOVA, TIME, F(1,21) = 4.9, *p* = 0.038, BF_incl_ = 7.1, η²p = 0.19; Fig. 2b), (ii) similar reduction in the hand contralateral (-61.6%) and in the hand ipsilateral (–63.8%) to the stimulated hemisphere (TIME x SIDE, F(1,21) < 0.1, *p* = 0.86, BF_incl_ = 0.28, η²p = 0.001; Fig. 2b), (iii) inconclusive evidence of absence of differences between stimulation of the right and stimulation of the left hemisphere (TIME x SIDE x STIMULATION, F(1,21) = 1.0, *p* = 0.32, BF_incl_ = 0.51, η²p = 0.046), (iv) moderate evidence of absence of an effect 15 min after tSMS compared to baseline (TIME, F(1,21) < 0.1, *p* = 0.93, BF_incl_ = 0.22, η²p < 0.001; Supplementary Fig. 1b).

Kinetic tremor 5 min after tSMS compared to baseline (secondary outcome) displayed inconclusive evidence of reduction (three-way ANOVA, TIME, F(1,25) = 3.6, *p* = 0.068, BF_incl_ = 1.2, η²p = 0.13; TIME x SIDE, F(1,25) = 0.6, *p* = 0.43, BF_incl_ = 0.30, η²p = 0.025; TIME x SIDE x STIMULATION, F(1,25) = 1.6, *p* = 0.21, BF_incl_ = 0.38, η²p = 0.061; Fig. 2c). Similar results were obtained at 20 min after tSMS compared to baseline (three-way ANOVA, TIME, F(1,21) = 4.3, *p* = 0.050, BF_incl_ = 0.85, η²p = 0.17; Supplementary Fig. 1c).

All mean values for postural tremor amplitude and frequency, rest tremor amplitude and kinetic tremor are represented in Table 2.

**Table 2:** Primary and secondary outcomes

	Baseline	Immediately after tSMS	15 min after tSMS
Postural tremor amplitude (log_10_ a.u.) *Contralateral to stim. hemisphere*	3.0 ± 1.1	2.6 ± 1.2	2.8 ± 1.2
Postural tremor amplitude (log_10_ a.u.) *Ipsilateral to stim. hemisphere*	2.8 ± 1.0	2.5 ± 1.3	2.6 ± 1.1
Postural tremor frequency (Hz) *Contralateral to stim. hemisphere*	5.5 ± 0.8	5.4 ± 0.9	5.5 ± 0.8
Postural tremor frequency (Hz) *Ipsilateral to stim. hemisphere*	5.4 ± 0.8	5.4 ± 0.8	5.5 ± 0.8
Rest tremor amplitude (log_10_ a.u.) *Contralateral to stim. hemisphere*	1.7 ± 1.0	1.3 ± 0.8	1.6 ± 0.9
Rest tremor amplitude (log_10_ a.u.) *Ipsilateral to stim. hemisphere*	1.9 ± 1.0	1.4 ± 0.9	1.9 ± 1.0
Kinetic tremor scores *Contralateral to stim. hemisphere*	6.1 ± 2.9	5.6 ± 2.4	5.7 ± 2.8
Kinetic tremor scores *Ispilateral to stim. hemisphere*	6.1 ± 2.8	5.7 ± 2.7	5.9 ± 3.1

Values represent the average of left and right tSMS patient group separating the effects by stimulated hemisphere (real tSMS) and sham hemisphere. Kinetic tremor was rated based on the drawing item from CRST Part B. Sample sizes for postural and rest tremor values, *n* = 23; samples sizes for kinetic tremor, *n* = 27 (except for 15 min after tSMS, *n* = 23).

*tSMS* transcranial static magnetic field stimulation, *stim* stimulated.

### Safety

No tSMS-related side effects were observed during and after the procedure.

## Discussion

Our study suggests that one session of 30-min tSMS applied to the primary motor cortex of ET patients is safe and may reduce postural and rest tremor at least in the short term, with comparable effects observed in both the contralateral and ipsilateral hand relative to the stimulated hemisphere and no effect on tremor frequency. The effects appeared to subside within 15 min, indicating a return to baseline tremor levels. The effects on kinetic tremor were inconclusive.

As the primary outcome, postural tremor amplitude showed moderate evidence of reduction compared to baseline when measured immediately after 30-min tSMS application. Unexpectedly, the average tremor reduction was similar in both the hand contralateral and ipsilateral to the stimulated hemisphere. The mechanism by which unilateral cortical stimulation mat improve bilateral postural tremor remains to be elucidated. Earlier studies in ET patients have indicated that central oscillators in the right and left hemispheres are not completely independent from one another, suggesting that synchronized oscillations might occur by interhemispheric coupling via the corpus callosum^42^. Therefore, we propose that tSMS likely disrupted the tremor-generating network within the stimulated motor cortex, which may also have led to changes in the contralateral hemisphere via corpus callosum connections^24^, resulting in a bilateral improvement of tremor. Previous investigations using inhibitory TMS protocols such as continuous theta-burst stimulation (cTBS) over the motor cortex reported reductions in tremor amplitude in ET patients through unilateral hemispheric stimulation^43–45^. Yet, in these works the possibility of a bilateral effect has not been explicitly studied, i.e. bilateral tremor improvement cannot be ruled out.

At the pathophysiological level the involvement of the cerebellum^46–51^ and the thalamus^52–54^ in ET has been widely accepted, whereas the contribution of the cerebral cortex has long been disputed. A disruption of the tremor-generating network induced by tSMS via motor cortex stimulation supports the notion that the motor cortex contributes to the generation or sustainment of tremor in ET^37–40^. Supporting this, a recent study using simultaneous intracranial thalamic recordings and magnetoencephalography (MEG) demonstrated that tremor episodes are characterized by increased oscillatory coupling between the thalamus, cerebellum, and primary sensorimotor cortex at individual tremor frequency, with coupling strength correlating with tremor amplitude^55^. Accordingly, several MEG and EEG investigations have observed a clear representation of the tremor rhythm in the cortical activity of ET patients during tremor activation^37–39,42^, with evidence that these tremor-related correlations are transmitted from supplementary motor areas or the primary motor cortex to the muscle^40^. Other studies confirmed that the observed rhythmic representation characterizes a genuine cortical output rather than just being a feedback-triggered phenomenon^39,40^. Complementing this, TMS-based studies have reported altered cortical excitability in ET patients, but supporting a normal GABAergic function^56^, while others showed modulation of cortical circuits in response to pharmacologic treatments such as primidone and propranolol, suggesting a central mechanism of action involving both GABA_A_ and GABA_B_ pathways^57^. Altogether, this suggests a pathophysiological role of the motor cortex in ET being part of the central tremor-generating network that involves most likely the cerebello-thalamo-cortical loop^34,36,55,58^. Recent evidence further supports a broader role of the motor cortex in ET beyond tremor generation. Reduced corticospinal excitability and impaired motor cortex plasticity have been shown to correlate with slower finger-tapping performance in ET patients, suggesting that motor cortex dysfunction may contribute not only to tremor but also to bradykinesia-like features and deficits in voluntary motor control^59^.

Since we obtained a bilateral improvement in tremor with unilateral tSMS, the main limitation of our experimental protocol is the absence of a true sham stimulation (i.e., a group receiving only sham stimulation). The absence of a true sham stimulation is a common strategy in pilot studies exploring the potential of an NIBS technique for a new indication. However, in this case, the bilateral reduction in tremor amplitude − despite unilateral stimulation—raises the possibility that the effect may be partially attributable to nonspecific factors such as suggestion or emotional modulation^60,61^. To better control for these potential confounds and strengthen causal inferences, future studies should employ randomized, sham-controlled, and double-blind designs. Furthermore, incorporating neurophysiological assessments—such as TMS-derived measures of cortical excitability − would allow for a more direct evaluation of the modulatory effects of tSMS on motor cortical circuits, thereby reinforcing mechanistic claims. Another methodological limitation concerns the potential residual effects of anti-tremor medications. Although all assessments were conducted in the morning before the daily dose, variability in pharmacokinetics, particularly for drugs with longer half-lives, may have led to incomplete washout. Therefore, a pharmacological confounding effect cannot be entirely excluded.

In our study, secondary outcome measures revealed several observations. Postural tremor frequency showed moderate evidence of no change. Interestingly, rest tremor amplitude showed a notable reduction immediately after tSMS, with similar effects in both hands. At 15 min post-tSMS, there was no clear evidence of a change in postural tremor amplitude compared to baseline, suggesting that tremor levels returned toward baseline values. Similarly, rest tremor amplitude also returned to baseline 15 min after tSMS, indicating no prolonged improvement. Finally, for kinetic tremor, the evidence of reduction 5 min post-tSMS was inconclusive.

A priori, we did not strongly expect to improve tremor frequency with tSMS of the motor cortex. In fact, several previous studies were already unable to modulate tremor frequency applying repetitive TMS protocols in different tremor syndromes^44,45,62^. Similarly, investigations using sensory electrical stimulation to modulate tremor were not able to modulate tremor frequency in ET patients^63,64^. Indeed, tremor frequency is knowingly influenced by only a few factors, primarily age^65,66^ and alterations in membrane excitability^67^. Thereby, in our study inhibitory tSMS of the motor cortex might reduce the oscillatory activity in the tremor network, but without altering its inherent frequency.

Since its initial description, the presence of rest tremor in ET patients has remained a controversial issue^68^. Anyway, the occurrence of ET with rest tremor is relatively common, with studies reporting that rest tremor can be present in up to 72.9% of ET cases^69^. Our findings showed that rest tremor amplitude was, as expected, at least one order of magnitude smaller than postural tremor amplitude, but it was similarly responsive to tSMS. The observed synchronous effect of tSMS on both postural and rest tremor is consistent with the fact that both types share at least partially a common underlying pathophysiology in ET characterized by alterations of the cerebello-thalamo-cortical loop^70^. This is further supported by studies performed in Parkinson’s disease patients, showing that both tremor types respond similarly to dopaminergic treatment^71,72^ and present cross-interactions^72^.

Neither postural nor rest tremor amplitude showed prolonged improvement 15 min after tSMS application, indicating a return to baseline tremor levels. This outcome is not surprising, considering that this pilot study involved only a single 30-min session. Previous research has shown that neurophysiological changes in corticospinal excitability induced by tSMS can persist for up to 30 min following a single 30-min session^22,26,27^. However, effective and prolonged clinical improvement often necessitates multiple sessions of transcranial stimulation. To achieve a greater impact on clinical efficacy in ET patients, multiple tSMS sessions would likely be required. This would also allow for the assessment of additional functional outcomes such as daily living activities, quality of life, and patient satisfaction. Importantly, the portable and user-friendly nature of tSMS makes it particularly appealing for home-based treatments^13,15^. Consequently, this approach could enable long-term, non-invasive treatment options for patients with ET, reducing the need for daily hospital visits that are often necessary with other NIBS techniques.

Despite the observed reduction in postural and rest tremors, our pilot study did not show convincing improvement in kinetic tremor. This discrepancy might be attributed to the higher sensitivity of accelerometry in detecting tSMS-induced changes in postural and rest tremors compared to the less objective scoring of Archimedes spirals. To address this, employing accelerometry, digital graphic tablets^73–75^, optoelectronic systems^76,77^, or motion transducers^78,79^ could provide a more precise quantification of kinetic tremor. It is also conceivable that the effect of tSMS was already starting to decay at the assessment of kinetic tremor, which started with a five-minute delay with respect to postural tremor. Nevertheless, multiple sessions of tSMS might result in improvements in kinetic tremor, regardless of the evaluation method used. One limitation of our study is that our findings are constrained to specific motor features of ET patients, particularly the kinetic tremor assessed solely by Part B of the CRST. Future research should include a more comprehensive clinical evaluation of tremor before and after tSMS interventions to better assess the full range of effects.

With respect to clinical safety, a single session of 30-min tSMS of the motor cortex was feasible and well tolerated in ET patients, with no tSMS-related adverse effects to be reported. These findings affirm the safety of tSMS^80^. Decades of clinical use of MRI, which involves static magnetic fields ranging from 1.5 to 3 Tesla and even up to 7 Tesla, further support this safety. These MRI fields are an order of magnitude stronger than those used in tSMS ( < 200 mT at the cortical level^81^), reinforcing the safety of brain exposure to static magnetic fields.

Overall, the results of this study suggest that tSMS may improve tremor in ET. These findings indicate that tSMS is a promising therapeutic approach for the treatment of ET, which should be confirmed in randomized controlled trials with multiple sessions of tSMS.

## Methods

### Trial design

We conducted a randomized, double-blind pilot study testing the hypothesis that tSMS of the primary motor cortex reduces tremor in patients with ET. The study was performed at HM CINAC, Hospital Universitario HM Puerta del Sur, Móstoles, Madrid from May 2017 to September 2018. The study was registered at ClinicalTrials.gov (NCT03780426) on December 19, 2018. No important changes to methods were performed after study commencement.

### Participants

Thirty patients with medically-refractory ET^82^, diagnosed by a neurologist specialized in movement disorders (DU and LVD), were enrolled in the study (14 females; mean age: 66.0 ± 14.3 years). Medically-refractory ET was defined as a persistent, functionally impairing tremor that did not show satisfactory response after at least two adequate trials of therapeutic medications, including at least one first-line agent such as propranolol or primidone. Out of all patients, 14 were receiving a single anti-tremor medication, while 9 were treated with a combination of anti-tremor drugs. Four patients were not on any anti-tremor treatment at the time of study enrollment, despite previous adequate therapeutic attempts. Detailed information on average dosages and group-wise distribution of all anti-tremor medications is shown in Supplementary Table 1. Exclusion criteria were MRI-incompatible metal objects in the body (e.g. cardiac pacemakers), evidence of other prominent neurological signs, such as dystonia, ataxia, parkinsonism, or dementia, and the presence of major psychiatric disorders. The presence of axial tremor was not considered an exclusion criterion as long as it occurred in combination with bilateral upper limb action tremor, in accordance with the diagnostic criteria for ET. All medications were required to remain stable at least one month before the beginning of participation. To minimize any potential confounding effect due to pharmacological therapy, all assessments were performed consistently in the morning, before patients took their daily medication dose. Three of these patients were excluded before undergoing the assigned procedure because they met exclusionary criteria. As predefined in the protocol and statistical analysis plan, only the 27 patients (13 females; mean age: 66.9 ± 12.1 years) who underwent the study procedure were included in the modified intention-to-treat analysis. Of these, 23 completed the full study protocol, which included both tremor recording and clinical assessments. A CONSORT flow diagram is provided in Fig. 1. The remaining four patients received only the clinical assessment. The study was performed according to the Declaration of Helsinki and approved by the local Ethics Committee at HM Hospitales, and all participants signed informed written consent before tSMS session.

### Interventions

**Fig. 3:**
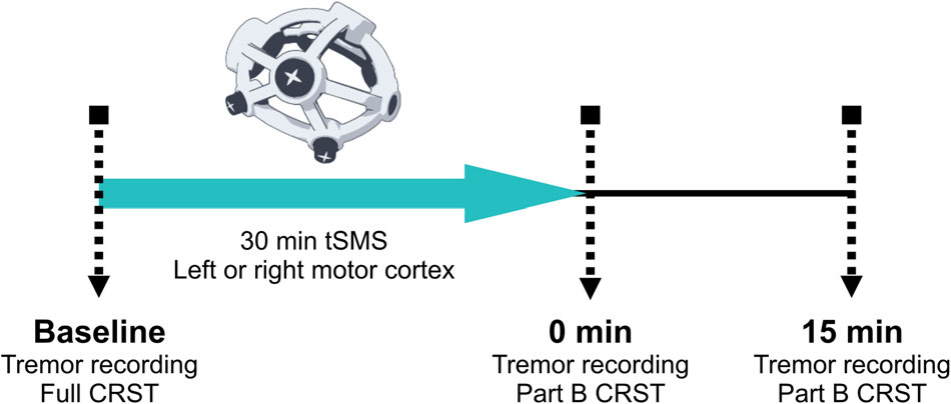
Experimental design. Each patient underwent one 30-min tSMS session of either the left or right motor cortex (sham tSMS was placed on the contralateral side to the stimulated side). At baseline the full CRST and tremor recording through accelerometry were assessed. After the tSMS intervention, tremor recording was performed through accelerometry for 5 min, followed by the assessment of kinetic tremor using Part B of the CRST. This sequence was repeated both immediately after and 15 min after finishing the tSMS intervention. tSMS transcranial static magnetic field stimulation, CRST Clinical Rating Scale for Tremor.

Real tSMS was delivered with a cylindrical neodymium magnet (grade N45) of 45 mm diameter and 30 mm of thickness, with a weight of 360 g (MAG45r; Neurek SL, Toledo, Spain; see ref. ^28^ for estimations of the magnetic field along the axis), applied with south polarity pointing toward the left or right motor cortex (assigned by GF), over the representational field of the hand area (C3 or C4 according to the 10/20 system). A non-magnetic metal cylinder (sham), with the same size, weight and appearance of the magnet (MAG45s; Neurek SL, Toledo, Spain) was simultaneously applied over the motor cortex contralateral to the stimulated hemisphere. Real and sham magnets were held with an ergonomic helmet (MAGmv1.0; Neurek SL, Toledo, Spain; Fig. 3). The overall weight of the helmet was sustained with a flex arm equipped with a spring of equivalent force.

### Clinical outcomes

The primary outcome was the change from baseline in postural tremor amplitude in the hand opposite the tSMS-treated hemisphere, measured by accelerometry immediately after tSMS. The accelerometer was placed on the dorsal hand surface, with the Z-axis parallel to the hand’s axis and the Y-axis perpendicular to the palm. Two 100-second recordings were taken with arms outstretched. To avoid fatigue and record rest tremor, hands rested for 100 s between recordings. Accelerometer signals were band-pass filtered (2 Hz-2 kHz) and amplified (×1000; D360, Digitimer Ltd). Digital signals were collected via an analog-to-digital converter (CED 1401, Cambridge Electronic Design). A blinded investigator conducted postural and rest tremor assessment and analyses (DU).

The secondary outcome measured were the following: (i) change from baseline in postural tremor amplitude 15 min after treatment, as measured by accelerometry; (ii) change from baseline in rest tremor amplitude, as measured by accelerometry; (iii) change from baseline in postural tremor frequency, as measured by accelerometry; (iv) change from baseline in kinetic tremor, as assessed by the drawing part of CRST part B. The task required the patient to draw two Archimedes spirals (with less space available in the second) and a straight line between narrow confines, with each item rated on a scale from 0 to 4^83^. Two blinded investigators (MHGM and BFR) independently rated this task.

Safety was assessed by the examining neurologist (DU), blind to the treatment, who completed physical and neurological evaluations for each session. The Standard Code of Federal Regulation definitions for serious adverse events were considered when evaluating adverse events.

### Experimental procedures

The following procedure was performed for each patient (Fig. 3). At baseline the full CRST score was assessed in each patient. Tremor recording by accelerometry and tremor evaluation of part B of the CRST were assessed before, immediately after and 15 min after 30 min of motor cortex tSMS application. Note that part B CRST evaluation (kinetic tremor) was always performed after the accelerometer recordings, which lasted 5 min. Patients received tSMS of either the left hemisphere and contralateral sham (*n* = 14) or the right hemisphere and contralateral sham (*n* = 13). The patients were seated comfortably with their forearms placed on the armrest up to the wrist, leaving the hand freely movable. They were instructed to refrain from speaking and to remain awake while in a calm, relaxed state during the whole procedure.

### Sample size

We aimed for recruiting 30 patients, taking into account patients that do not meet inclusion criteria. Note that Bayesian evidential strength becomes reasonable starting from a sample size of 20 participants.

### Randomization

Patients were randomly assigned to receive real tSMS of either the left hemisphere (*n* = 14) or the right hemisphere (*n* = 13). Randomization was performed using coin flip (DU). Two helmets were used to ensure blinding.

### Blinding

All patients, caregivers, and investigators assessing outcomes were blind to the intervention assignment. Randomization codes were kept by an independent researcher and were only revealed after data analysis.

### Data analysis

Tremor recordings were analyzed by power spectral analysis to determine the dominant signal’s amplitude, expressed as the power or the main peak, and frequency peak using Spike2 software (Cambridge Electronic Design). For postural tremor, the mean of the two recordings available at each time point was used for the final statistical analyses. Power values were log-transformed to ensure homoscedasticity for the statistical analyses.

### Statistical analysis

All statistical analyses were performed with Bayesian statistics^84^ as implemented in JASP (version 0.14). Clinical comparisons between groups were performed with unpaired t-tests and reported using two-tailed Bayes factors (BF_10_). Log-transformed total power values of postural and rest tremor amplitude, as well as tremor frequencies and kinetic tremor scores were separately entered into a three-way mixed ANOVA with the following factors: TIME (baseline vs. after intervention) and SIDE (contralateral vs. ipsilateral hand) as within-subjects factors, and STIMULATION (left vs. right cortex) as between-subjects factor. Bayesian ANOVAs were conducted with default priors and random intercepts only, and effects are reported as the Bayes factor for the inclusion of a particular effect (BF_incl_), calculated as the ratio between the likelihood of the data given the model with vs. the next simpler model without the effect, comparing across matched models. Evidence in favor of alternative hypothesis (*BF* > *1*) or to the null hypothesis (*BF* < *1*) was described according to standard levels: anecdotal (*1/3* < *BF* < *3*), moderate *(* < *1/3 or* > *3*), strong *(* < *1/10 or* > *10*), very strong ( < *1/30 or* > *30*), extreme ( < *1/100 or* > *100*). Standard p-values of corresponding frequentist ANOVAs are also reported for completeness.

## Supplementary information

Supplementary Data 1

Supplementary information

Supplementary Table 1

## Data Availability

The dataset used for the analysis of the present study is provided within Supplementary Data 1.
