# Supplementary material for: Transcranial static magnetic field stimulation of the primary motor cortex in essential tremor: a randomized pilot study
Source: NPJ Parkinsons Dis. 2025 Nov 26;11:336. doi: 10.1038/s41531-025-01182-x (PMC12658149; doi:10.1038/s41531-025-01182-x)
Supplement: Supplementary file 2 — Supplementary information [file 41531_2025_1182_MOESM2_ESM.docx]

|  | **All patients** | **Left tSMS group** | **Right tSMS group** |
| --- | --- | --- | --- |
| Sample size, n | 27 | 13 | 14 |
| On anti-tremor treatment,  % number of patients:  One drug  Combination | 14 (51.9%)  9 (33.3%) | 6 (46.1%)  6 (46.1%) | 8 (57.1%)  3 (21.4%) |
| Medication usage,  % number of patients  Propanolol  Primidone  Others* | 15 (55.6%)  6 (22.2%)  10 (37.0%) | 10 (76.9%)  4 (30.8%)  3 (23.1%) | 5 (35.7%)  2 (14.9%)  7 (50.0%) |
| Propanolol dosage, mg ± SD n=15 | 54.0 ± 47.5 | 60.0 ± 55.8 | 42.0 ± 24.9 |
| Primidone dosage, mg ± SD  n=6 | 500.0 ± 223.6 | 500.0 ±288.7 | 500.0 ± 0.0 |

**Supplementary Table 1. Group-wise distribution and dosages of anti-tremor medications**

* Three patients treated with gabapentin (300–1200 mg), 3 with clonazepam (1–2 mg), 2 with topiramate (175 mg), 1 with zonisamide (100 mg), and 1 with phenobarbital (30 mg).
